# Genetic Diversity and Population Structure of the Chinese Three-Keeled Pond Turtle (*Mauremys reevesii*)

**DOI:** 10.3390/ijms26125614

**Published:** 2025-06-11

**Authors:** Chenyao Zhou, Haoyang Xu, Haiyang Liu, Jipeng Li, Wei Li, Xiaoyou Hong, Chen Chen, Liqin Ji, Xinping Zhu, Bo Zhao, Xiaoli Liu

**Affiliations:** 1 School of Fishery, Zhejiang Ocean University, Zhoushan 316000, China; zhouchenyao@zjou.edu.cn (C.Z.); zhuxinping_1964@163.com (X.Z.); 2 Key Laboratory of Tropical and Subtropical Fishery Resources Application and Cultivation, Ministry of Agriculture and Rural Affairs, Pearl River Fisheries Research Institute, Chinese Academy of Fishery Sciences, Guangzhou 510380, China; 18457173557@163.com (H.X.); hyliu@prfri.ac.cn (H.L.); m240150622@st.shou.edu.cn (J.L.); liwei@prfri.ac.cn (W.L.); hongxiaoyou1216@163.com (X.H.); chenchen3729@163.com (C.C.); goofyji@126.com (L.J.); 3 College of Life Science and Fisheries, Shanghai Ocean University, Shanghai 201306, China

**Keywords:** *Mauremys reevesii*, population genomics, population structure, whole-genome resequencing

## Abstract

To investigate the genetic diversity and structure of farmed Chinese three-keeled pond turtles (*Mauremys reevesii*), we performed whole-genome resequencing on 238 individuals from eight farms across six Chinese regions. Genetic diversity indices (nucleotide diversity π, inbreeding coefficient *F_HOM_*, polymorphism information content PIC, observed heterozygosity *Ho*), principal component analysis (PCA), phylogenetic reconstruction, and population structure analysis were integrated. The results revealed that the Guangdong Maoming (MM) and Anhui Wuwei (WW) populations exhibited the highest genetic diversity (MM: PIC = 0.149, *Ho* = 0.299; WW: PIC = 0.144, *Ho* = 0.287), while the Guangdong Huizhou (HZ) and Hunan Changhan (CH) populations showed the lowest diversity due to elevated inbreeding coefficients (HZ: *F_HOM_* = 0.043; CH: *F_HOM_* = 0.041). Low genetic differentiation (*F_st_* = 0.00043–0.04758) indicated limited population divergence. However, PCA and phylogenetic analysis demonstrated that MM and Guangxi Pingxiang (PX) populations formed distinct genetic clusters, suggesting that management differences might contribute to their genetic uniqueness. Admixture analysis identified *K* = 2 (based on the lowest cross-validation error) as the optimal ancestral cluster number, with MM and PX populations displaying admixed genetic backgrounds while others showed homogeneous compositions. Conservation priorities should focus on preserving MM and PX’s unique genetic resources, introducing genetic material to high-inbreeding populations, and establishing interregional breeding networks. This study provides genomic insights for germplasm conservation and sustainable utilisation of *M. reevesii*.

## 1. Introduction

The Chinese three-keeled pond turtle (*Mauremys reevesii*) is a member of the family Geoemydidae, and is widely distributed across China, the Korean Peninsula, and Japan [1,2]. In Chinese culture, the turtle is regarded as an auspicious symbol due to its longevity, which is perceived as a quality that is conducive to the attainment of good fortune [3]. Furthermore, the Chinese three-keeled pond turtle is a rich source of amino acids, unsaturated fatty acids, essential minerals, and trace elements [4]. It has been employed in traditional Chinese medicine for centuries, with the main pharmacological effects being the replenishment of blood and vital energy, the tonification of the kidney, and the promotion of fertility, as well as the treatment of coughs and dysentery [5]. These properties have been extensively documented in ancient texts, including Shennong’s *Classic of the Materia Medica* and *Compendium of Materia Medica*, as well as the *Pharmacopoeia of the People’s Republic of China* [6,7,8]. Furthermore, the turtle’s ornamental value is significant, particularly in the case of male turtles that have been selectively bred to have a black body, which has led to the colloquial designation of “ink turtle” [9,10]. In recent years, the overexploitation and destruction of habitats have resulted in a significant decline in the natural population of the turtles, which are currently classified as “endangered” on the IUCN Red List of Threatened Species [11].

In order to further the conservation and development of turtle germplasm resources, breeders have been engaged in the practice of artificial breeding and the domestication of turtles since the 1970s [12]. This involves using sexually mature individuals from wild resources and rearing and breeding the offspring in ponds or in conservatories [13]. As of 2023, the number of turtle hatchlings has reached about 800 million with a production of about 30,000 tonnes (data from the First National Census of Aquaculture Germplasm Resources in China, unpublished). Artificial breeding can significantly increase the hatching rate of eggs and the survival rate of hatchlings, and once the population size has been increased through artificial breeding, some individuals can be systematically released into the natural habitat to replenish the wild population [14]. In addition, scientifically managed farms record genealogical information to avoid inbreeding and preserve the genetic diversity of the Chinese three-keeled pond turtle [15]. Finally, the farms provide data for research on turtle behaviour, reproductive ecology, and disease prevention and control. However, the current state of knowledge regarding the genetic composition of turtle breeding populations remains limited, with significant gaps in genome-wide diversity assessments and comprehensive evaluations of adaptive genetic variation in the existing literature. There is a paucity of systematic assessment of existing breeding populations in terms of genetic diversity maintenance and germplasm resource conservation, which poses a potential risk to the sustainability of the captive breeding system. Consequently, there is an urgent need to carry out genetic structure analysis to establish a standardised genetic resource management system, which could include (1) routine genetic diversity monitoring through the genotyping of breeding cohorts using molecular markers (e.g., microsatellites or SNPs); (2) the implementation of pedigree tracking software to minimise inbreeding coefficients; (3) structured breeding protocols ensuring optimal mate pairing based on kinship analysis; (4) the cryopreservation of gametes or somatic tissues from genetically underrepresented individuals; and (5) the periodic introduction of wild individuals to replenish genetic variation. Such measures would provide a theoretical foundation for scientifically robust conservation practices.

In a previous study, Zhu et al. investigated the genetic diversity of the *Chinemys reevesii* (=*M. reevesii*) by using random amplified polymorphic DNA (RAPD) techniques, and found that the Chinese three-keeled pond turtles were highly genetically diverse by analysing the genomic DNA of 24 individuals collected in Hubei Province using 20 primers [16]. The results of the genetic analysis of seven Chinese three-keeled pond turtles in Guangxi Zhuang Autonomous Region using RAPD technology demonstrated that the genetic diversity at the population level was abundant (polymorphism frequency of 66.29%), yet the individuals were more closely related and exhibited reduced genetic variability (average genetic distance index of 0.2092) [17]. In a further study, Zhang et al. 2010 employed microsatellite markers to assess the genetic diversity of seven turtle breeding populations. The findings indicated that all seven populations exhibited high polymorphism and substantial genetic variation [18]. Additionally, each population exhibited distinctive alleles, indicating that the diffusion of alleles between populations is subject to certain constraints. This information can serve as a valuable reference index for the selection of parents in breeding programmes [18]. Bu et al. 2019 assessed the structure of five captive populations of the Chinese three-keeled pond turtle based on 12 polymorphic microsatellite markers, and found that these five populations had moderate to high genetic differentiation and different inbreeding [19]. Consequently, the authors proposed that it is essential to breed new superior populations with high genetic diversity using different captive populations of this species [19]. Furthermore, phylogenetic analyses of the distribution and relationships of mitochondrial DNA *Cytb* gene sequences of East Asia populations have been conducted, indicating that this taxon is monophyletic. This finding suggests that they evolved from a single matriarchal lineage and underwent localised evolution following geographic migration and isolation in East Asia [20].

In recent years, whole-genome sequencing technologies have been progressively applied to genetic studies of turtles and tortoises. For example, researchers at the Kunming Institute of Zoology successfully assembled a chromosome-level high-quality genome (2.24 Gb) of a female Swinhoe’s softshell turtle (*Rafetus swinhoei*) by integrating Nanopore long-read sequencing, BGI-seq short-read sequencing, and chromosome conformation capture (Hi-C) techniques. This revealed its ZZ/ZW sex determination system. Analyses identified positive selection in genes related to autophagy and DNA damage response, explaining the genetic basis for its longevity and large body size, while the absence of tooth-forming genes clarified the genetic foundation of its edentulous phenotype [21]. Comparative genomics with the Chinese softshell turtle (*Pelodiscus sinensis*) showed that the two species diverged 54.4 million years ago. Population history analyses indicated a continuous decline in the effective population size of *R. swinhoei*, with extremely low genetic diversity and heterozygosity, providing molecular evidence for optimising field surveys and conservation strategies [21]. Liu et al. 2022 utilised PacBio long-read sequencing, Illumina, and Hi-C technologies to construct a high-quality genome assembly for the Asian yellow pond turtle (*M*. *mutica*) [22]. Comparative genomic analyses revealed that the lizard–snake–tuatara lineage diverged from the bird–crocodilian–turtle lineage approximately 267–312.3 million years ago [22]. Additionally, numerous positively selected genes were enriched in calcium-signalling pathways and neuroactive ligand–receptor interactions, which may participate in temperature-dependent sex determination regulation, offering potential targets for sex-controlled breeding [22]. Similarly, Zeng et al. 2024 identified 21 female-specific SNP markers through whole-genome resequencing and Sanger sequencing in *P*. *sinensis* [23]. Nineteen of these markers were located within the *Znrf3* gene, which is associated with testis determination in mammals. This gene exhibited female-biased expression in *P*. *sinensis*, laying the foundation for the functional validation of sex-related genes [23].

In comparison to single-gene, RAPD, or microsatellite molecular markers, whole-genome resequencing offers a number of advantages. Such advantages include the elimination of the necessity to measure the length of amplified fragments, high genetic stability, high distribution density in the genome, and the ability to detect structural variations and selective signals across the entire genome [24]. Consequently, whole-genome resequencing is a frequently employed technique in the fields of population evolution, genetic map construction, and functional gene mining research, particularly demonstrating unique strengths in deciphering the genetic basis of complex traits and reconstructing population divergence histories [25].

The present study involved the collection of the eight primarily cultured strains of *M. reevesii* from China, which were then subjected to genetic analyses. The objective of this study was to elucidate the genetic status of the primary germplasm resources of the Chinese three-keeled pond turtle. By integrating high-throughput data at the whole-genome scale, we not only validated conclusions from previous studies based on traditional molecular markers, but also uncovered deeper genetic structures undetected by microsatellite and mitochondrial analyses. The findings of this study offer valuable insights that can inform the scientific management of aquatic resources and the selective breeding and genetic improvement of *M. reevesii*.

## 2. Results

### 2.1. Quality of Resequencing Data and Alignment with Reference Genome

In order to investigate the genetic diversity and population structure of the eight populations, 238 individuals of *M. reevesii* were collected for genome resequencing. The total sequencing bases were 7893.32 Gb, the average sequencing bases were 33.17 Gb, and the average depth was 12.68X. The amount of sequencing data and filtering results are shown in Table 1. Among them, the average value of Q30 was 90.86~93.28%, and the average value of GC content was in the range of 44.06~44.35%, which indicated that the samples were built with good quality (Q30 value ≥ 90%, i.e., the base identification error rate was ≤ 0.1%), which was in line with the standard for genome resequencing (Table 1).

The results of the comparison of the resequencing data with the reference genome (NCBI accession: GCA_016161935.1) showed that the mean number of sequences obtained after quality control was 211,544,440~234,143,127, the mean genome comparison rate was 98.54~99.87%, the average sequencing depth was 11.83X~13.37X, and the average coverage was 92.30~92.66%. The alignment rate reflects the similarity between the sequencing data of the samples and the reference genome, indicating that the alignment results are normal and can be used for subsequent SNP locus screening and genetic structure analysis (Table 1).

### 2.2. Detection and Statistical Analysis of SNP Variants

The statistical analysis of the SNP loci showed that the MM group had the highest number of SNP loci at 18,170,044. It was followed by the CH group with 15,505,440 SNP loci, and the PX group with 15,451,204 SNP loci. The group with the least SNP loci was the HZ group with 14,021,985 loci. The other groups had between 14.11 million and 14.5 million SNP loci (Table 2).

When comparing the average percentage of homozygous (*Ho*) and heterozygous (*He*) synonymous SNPs across eight *M. reevesii* populations, the HZ population showed the highest homozygous SNP percentage (33.73%) with the corresponding lowest heterozygous SNP level (66.27%). This was followed by ZQ (*Ho* = 33.35%) and YY (*Ho* = 32.91%) populations. In contrast, the MM population exhibited the lowest homozygous SNP percentage (26.94%) accompanied by the highest heterozygous SNP proportion (73.06%). Other populations displayed intermediate values, with PX (*He* = 68.55%) and WW (*He* = 68.09%) showing relatively higher heterozygous SNP percentages (Table 2).

### 2.3. Population Genetic Diversity

A comparative analysis of the nucleotide polymorphisms of the groups showed that the PX group had the highest nucleotide polymorphism, π = 0.0000379257, followed by the WW group with π = 0.000037696. The lowest nucleotide polymorphism was found in the MM group, with a π value of 0.0000358555. The group with the lowest coefficient of inbreeding was MM with an *F_HOM_* of −0.057 ± 0.081, followed by WW with an *F_HOM_* of −0.015 ± 0.112. The group with the highest inbreeding coefficient was HZ with an *F_HOM_* of 0.043 ± 0.037, followed by CH with an *F_HOM_* of 0.041 ± 0.057. The group with the highest polymorphic information content was MM with a PIC value of 0.149 ± 0.011, followed by WW with a PIC value of 0.144 ± 0.016, and the group with the lowest population polymorphic information content was HZ. The group with the highest observed heterozygosity was MM with an *Ho* value of 0.299 ± 0.023, followed by the WW group with an *Ho* value of 0.287 ± 0.032; the group with the lowest observed heterozygosity was HZ with an *Ho* value of 0.270 ± 0.011, followed by the CH group with an *Ho* value of 0.271 ± 0.016 (Table 3). The results of the combined group polymorphic information content and observed heterozygosity show that among the eight groups, the MM group and the WW group have the highest genetic diversity; the HZ group and the CH group have the lowest genetic polymorphisms and the inbreeding coefficients also support this result, with the highest inbreeding coefficients in HZ and CH, further indicating that some degree of inbreeding exists within these two populations.

Pairwise multilocus *F_st_* analyses of the eight geographical groups of *M. reevesii* were calculated according to the allele frequencies of each locus (Table 4). The genetic differentiation between the MM and JS groups was the greatest (0.04758), while that between the HZ and ZQ groups was the smallest (0.00043) (Table 4). *F_st_* < 0.05 indicated a low level of genetic differentiation [26]. The results suggested that the PIC among different populations was low, the degree of genetic differentiation between populations was relatively low, and the provenance of *M. reevesii* was relatively homogeneous.

### 2.4. Phylogenetic Relationship Analysis

**Figure 1:**
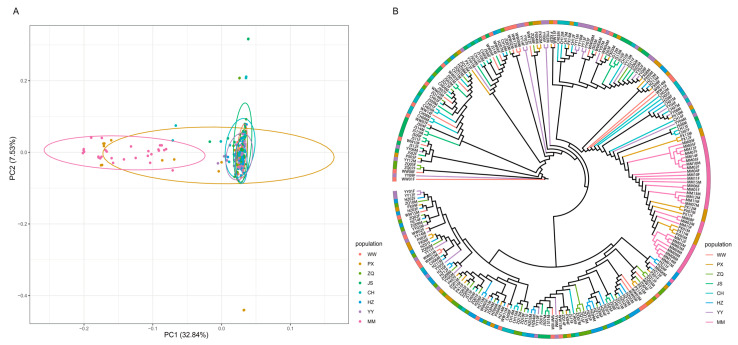
Results of principal component analysis (**A**) and phylogenetic tree construction based on the neighbour-joining method. Each branch in the picture is a sample, and each source has a colour (**B**).

Genetic background similarity and clustering among the eight populations was explored by principal component analysis (PCA) based on the first two principal components (PC1 and PC2), where PC1 explained 32.84% of the total variance and PC2 explained 7.53%. The MM population (pink) was distributed on the leftmost side of the PC1 axis and the PX population (brownish-yellow) was in the neighbourhood (Figure 1A), indicating that both were significantly genetically differentiated from other populations (WW, ZQ, YY, CH, HZ, JS) in the PC1 direction; this result was consistent with the conclusion of the genetic diversity analysis. A phylogenetic tree analysis showed that the MM and PX groups formed independent branches (Figure 1B), which was consistent with the PCA results and supported their genetic uniqueness, while the rest of the groups clustered in adjacent branches, suggesting that they may have a relatively recent co-evolutionary history.

### 2.5. Genetic Structure Analysis

**Figure 2:**
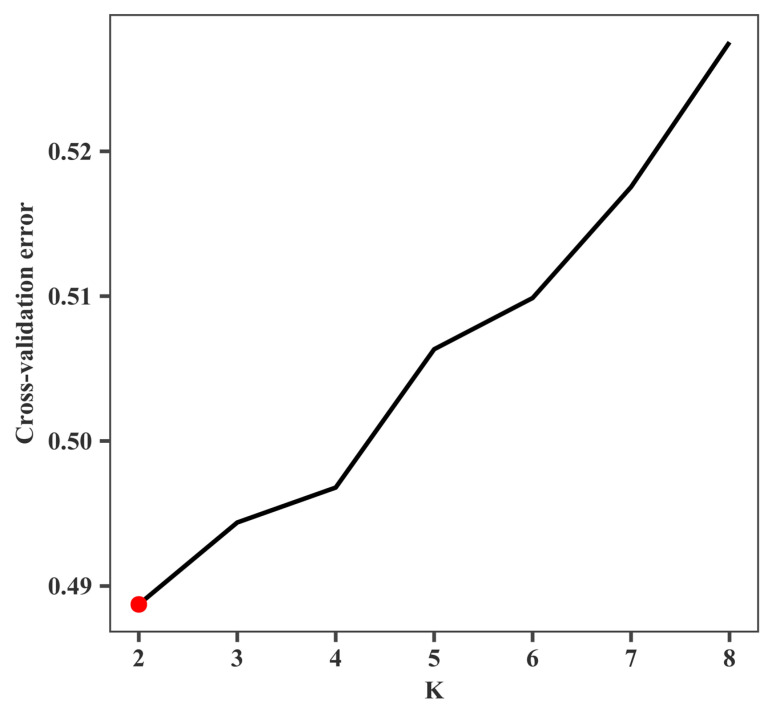
Distribution of cross-validation error values with different *K* values. Red dot indicates the *K* value with minimum cross-validation error, suggesting the best model complexity.

**Figure 3:**
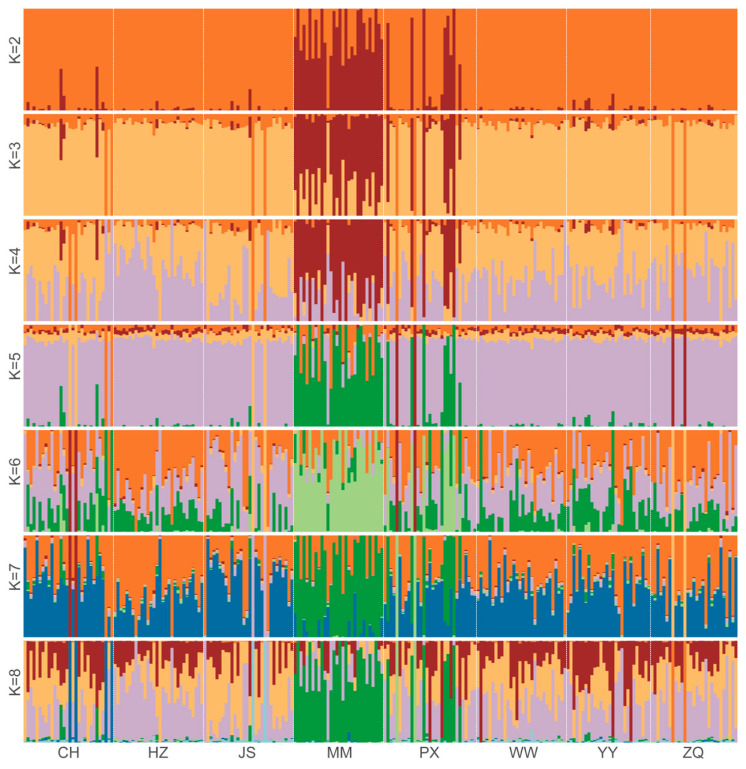
Population structure with each *K* value (*K* = 2–8).

As demonstrated in Figure 2, the genetic components were resolved for all individuals of the eight populations based on the assumed number of ancestors (*K* = 2–8). The clustering results were cross-validated, and the error value reached its lowest level when *K* = 2. Consequently, it was determined that *K* = 2 was the optimal number of clusters, and therefore, the species could be classified into two ancestral genetic components (Figure 2). As demonstrated in Figure 3, each column in the figure represents an individual, and the length of its different coloured segments is indicative of the proportion of the corresponding ancestor in the genome of the individual (one colour represents one ancestral population). The existence of genetic admixture between two populations is indicated by the overlap of their respective colour distributions. Specifically, the MM and PX populations exhibited analogous colour composition (predominantly red and orange), indicating a probable shared genetic origin. In contrast, the remaining populations (WW, ZQ, YY, CH, HZ, JS) displayed a single colour dominance (orange), suggesting a homogeneous genetic composition and an independent ancestral origin. The result is consistent with the findings of the genetic differentiation analysis and the phylogenetic tree.

## 3. Discussion

Maintaining the right level of population genetic diversity is a prerequisite for achieving the sustainable use of germplasm resources [27]. In this study, the level of genetic diversity of eight breeding populations of the Chinese three-keeled pond turtle was assessed using the dual indices of polymorphic information content (PIC) and observed heterozygosity (*Ho*). The genetic diversity of the MM (PIC = 0.149, *Ho* = 0.299) and WW (PIC = 0.144, *Ho* = 0.287) populations was found to be higher than that of the other populations, whereas the genetic diversity of the CH population was found to be lower (PIC = 0.136, *Ho* = 0.271). HZ populations exhibited the lowest genetic diversity (PIC = 0.135, *Ho* = 0.270), a discrepancy that may be associated with its germplasm resource management strategy. The MM population, as a provincial original breeder farm, exhibited a high proportion of heterozygous single-nucleotide polymorphisms (73.06%) and a negative inbreeding coefficient (*F_HOM_* = −0.057), suggesting that its effective population size was larger or that there was gene flow input to avoid inbreeding decline. Conversely, the elevated inbreeding coefficients (*F_HOM_* = 0.043 and 0.041) observed in the HZ and CH populations may be attributable to protracted closed breeding or the utilisation of shared parental resources, which has potentially led to a diminution in genetic diversity. Of particular concern is the long-term viability of populations with elevated inbreeding coefficients like HZ and CH. Sustained inbreeding increases the risk of inbreeding depression, reducing fitness traits such as reproductive success, disease resistance, and adaptive capacity [28]. Over generations, this genetic erosion could heighten extinction vulnerability, especially when confronted with environmental stressors or disease outbreaks [29]. It is important to note that, despite the *F_st_* values of all populations being lower than 0.05, indicating an overall low degree of genetic differentiation, the differentiation values of the MM population from the other populations (*F_st_* = 0.02500–0.04758) were close to the threshold of intermediate differentiation. This suggests that they may carry unique adaptive alleles that are worthy of priority conservation.

Principal component analysis (PCA) and phylogenetic topology were employed in conjunction to confirm that the MM and PX populations exhibited distinct genetic compositions (Figure 1). This differentiation may be attributed to two factors: firstly, restricted gene flow due to geographic isolation effects; and secondly, the historical influence of diversified breeding strategies. The PX population, as a provincial proto-generic farm, exemplifies this second factor; its higher level of nucleotide diversity (π = 0.0000379257) likely reflects a positive impact from the introduction of abundant wild germplasm resources in Guangxi Zhuang Autonomous Region. Furthermore, with regard to the genetic background, the PX and MM populations were bred by the same enterprise manager, which does not preclude the possibility that the MM and PX populations were initially one large population and had not yet accumulated enough genetic variation to result in significant differences. Admixture analyses further demonstrated that the MM and PX populations exhibited a mixed genetic component when *K* = 2 (Figure 2 and Figure 3), while the other populations [e.g., the present study set out to explore the hypothesis that the national original breeding farms (JS and WW)] presented a single genetic background. This was based on the assumption that this background was related to the strict genealogical management and limited germplasm exchange policy in national-level farms. This outcome aligns with the “positive correlation between management intensity and genetic homogeneity” identified by Wang (2014) in *Hyriopsis cumingii* [30]. This suggests that placing excessive reliance on a limited number of superior parents may intensify the risk of genetic homogenisation [30].

Despite the negligible overall genetic differentiation of the Chinese three-keeled pond turtle breeding populations (*F_st_* < 0.05), the genetic uniqueness of the MM and PX populations indicates the potential for these populations to harbour genetic variation adapted to specific environments. It is recommended that differentiation strategies be adopted in the conservation of germplasm resources. The implementation of in situ conservation of MM and PX populations and the limitation of the introduction of non-essential exogenous genes to maintain their uniqueness are imperative. For populations with high inbreeding coefficients, such as HZ and CH, introducing germplasm from other populations is crucial to enhance genetic diversity. Furthermore, the establishment of a transregional joint breeding network, and the utilisation of genome information to guide parental selection and to balance the production of traits is essential for optimisation and genetic diversity maintenance [31]. The present study concentrated on the breeding population and did not collect wild Chinese three-keeled pond turtles from the control group. This may have resulted in an underestimation of the role of captive breeding in shaping genetic structure. Furthermore, the mean sequencing depth of 12.68X, while adequate for SNP detection, exhibits a constrained capacity to resolve rare variants. It is recommended that future studies combine long read-length sequencing technology in order to gain a more in-depth understanding of the contribution of structural variants to population differentiation. In addition, the integration of data on environmental factors will facilitate the revelation of the molecular mechanisms of adaptive evolution.

## 4. Materials and Methods

### 4.1. Sample Collection

**Figure 4:**
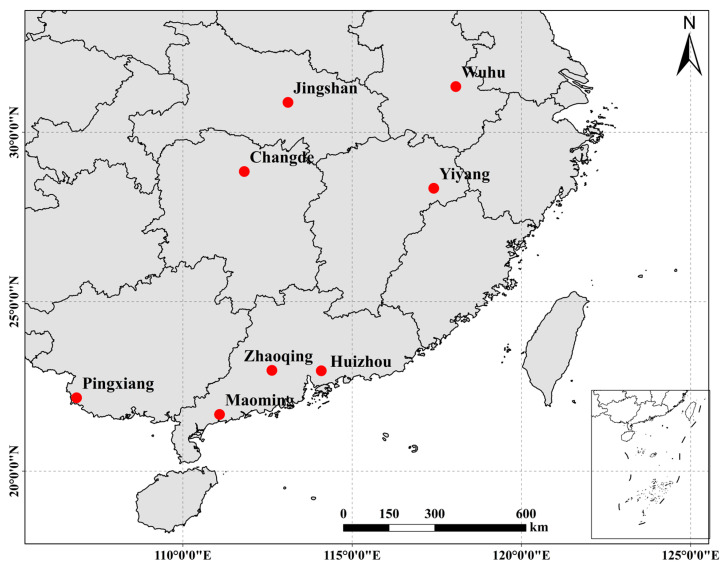
The distribution of the Chinese three-keeled pond turtle across its range of collection locations.

The turtles employed in this study were sourced from eight discrete farmed stocks, as follows: Anhui Wuwei (WW, *N* = 30), Hubei Jingshan (JS, *N* = 30), Jiangxi Yiyang (YY, *N* = 28), Guangdong Zhaoqing (ZQ, *N* = 30), Guangdong Huizhou (HZ, *N* = 30), Guangdong Maoming (MM, *N* = 30), Hunan Changhan (CH, *N* = 30), and Guangxi Pingxiang (PX, *N* = 30). A total of 238 samples were obtained from the eight sampling sites, as detailed in Figure 4 and Table 5.

### 4.2. Morphological Measurement

The weight of the Chinese three-keeled pond turtle was determined with an electronic balance with an accuracy of 0.1 g. The morphological traits of the Chinese three-keeled pond turtle, including dorsal carapace length, dorsal carapace width, body height, tail length, ventral carapace length, and ventral carapace width, were quantified with vernier callipers with an accuracy of 0.1 mm.

### 4.3. DNA Extraction and DNA Library Construction

In consideration of the regenerative properties of turtle toenails, a non-invasive sampling method involving sterile surgical scissors was employed to collect 1–2 mm distal segments of the third phalanx toenails from Chinese three-keeled pond turtles. This protocol followed established guidelines for reptile tissue sampling [32], with three welfare safeguards: (1) pre-disinfection using 75% ethanol to minimise infection risks; (2) the manual restraint duration was strictly limited to under 3 min per specimen; (3) the immediate application of haemostatic powder post-sampling. All procedures were approved by the Animal Ethics Committee of Pearl River Fisheries Research Institute, Chinese Academy of Fishery Sciences (LAEC-PRFRI-2023-07-02). Post-release monitoring demonstrated a 100% survival rate with complete nail regeneration within 8 weeks. The samples were assigned sequential numbers according to the individual turtles and then immersed in anhydrous ethanol in 1.5 mL Eppendorf tubes. The samples were stored in a refrigerator at 4 °C for future use. Genomic DNA was then extracted using the Genomic DNA Extraction Kit (MGIEasy, Shenzhen Huada Intelligent Science and Technology Co., Ltd., Shenzhen, China). The concentration of genomic DNA was quantified using a Qubit fluorometer, and the purity of the genomic DNA was assessed through agarose gel electrophoresis at a concentration of 1%. The DNA samples were randomly fragmented using an ultrasonic high-performance processing system (Covaris, Woburn, MA, USA), and fragments of approximately 500 base pairs were obtained following the selection process. Subsequently, the DNA fragments were subjected to end repair, with an “A” base appended to the 3′ end and library junctions introduced at both ends. Subsequently, the library was separated from the single strand and cyclised, in accordance with the junction connection. Subsequently, the cyclised library was then subjected to rolling circle amplification (RCA), which resulted in the generation of DNA nano balls (DNBs). Thereafter, the resulting DNBs were subjected to sequencing on the aforementioned apparatus, in accordance with the requisite quality control procedures. Prior to sequencing, quality control procedures were rigorously implemented, including the validation of DNB size distribution via dynamic light scattering (DLS), quantification of DNB concentration using fluorescence-based methods (Qubit 4.0, Thermo Fisher Scientific, Waltham, MA, USA), and verification of fluorescent labelling efficiency through spectral calibration.

### 4.4. Whole-Genome Resequencing and Reference Genome Comparison

Each qualified DNA library was subjected to high-throughput sequencing on the UWGI BGI autonomous sequencing platform DNBSEQ. The raw image data obtained from sequencing was converted into raw data (raw reads) through base recognition. The SOAPnuke (v2.1.0) software [33] was employed to filter the raw reads, with the objective of removing splice contamination and low-quality reads, and thereby obtaining high-quality, pure data (clean data). The filtered clean reads were aligned to the reference genome (NCBI accession: GCA_016161935.1) using BWA-MEM2 (v2.2.1). The alignment results were output as SAM files, which were subsequently converted into sorted BAM files using SAMtools (v1.17) [34]. Subsequently, the comparison result files were processed using SAMtools, Picard tools, and ReSeqTools, with the objective of sorting, deduplicating, and adding IDs. Subsequently, only those reads with a mapQ value exceeding 30 were selected for further analysis [35].

### 4.5. SNP Locus Analysis

The process of variant detection was conducted utilising the HaplotypeCaller tool in conjunction with the Genome Analysis Toolkit (GATK) software (version 4.6.2.0) [36]. The resulting gvcf files were subsequently merged using the CombineGVCFs tool, and the vcf files were obtained through co-genotyping with the GenotypeGVCFs tool. The filtering parameters were as follows: QD ≥ 2.0, FS ≤ 60.0, MQRankSum ≥ −12.5, ReadPosRankSum ≥ −8.0, and StrandOddsRatio > 3.0. The SelectVariants tool was employed for the purpose of filtering the SNP variants within the entire set of variants, with the objective of obtaining a high-confidence SNP dataset. This was accomplished through the utilisation of the VariantFiltration tool, which was specifically designed for this purpose. The number of single-nucleotide polymorphisms (SNPs) and the proportion of heterozygous and pure SNPs relative to the reference genome were calculated for each sample based on the obtained SNP dataset [37].

### 4.6. Genetic Diversity Analysis

The PLINKv1.90 software [38] was utilised to calculate the expected heterozygosity (*He*), the observed heterozygosity (*Ho*), and the polymorphism information content (PIC). Using the VCFtools v0.1.17 software, the genetic differentiation index (*F_st_*) was calculated between populations, nucleotide diversity (π) within populations, and inbreeding coefficient (*F_HOM_*) with a sliding window of 100 kb and a step size of 10 kb, to assess the degree of genetic differentiation among different breeding groups [39].

### 4.7. Population Genetic Structure Analysis

Principal component analysis (PCA) was conducted using PLINK v1.90 software [38], and the initial two principal components of the calculated results were visualised using the R language v4.2.1 package ggplot2 for PCA. The Admixture software (v1.3.0) [40] was employed to ascertain the genetic structure of the population. Each K value was repeated five times, after which the Pophelper software (v2.3.1) [41] was employed to calculate the ΔK value and merge the results of multiple repetitions in order determine the optimal K value based on ΔK. The merged results were then used to illustrate the population structure composition using a bar plot. Phylogenetic trees were constructed with PHYLIP v3.69 software [42] based on the neighbour-joining (NJ) method using the p-distances model with 1000 bootstraps and visualised with FigTree v1.4.4 software.

## 5. Conclusions

This study provides a comprehensive assessment of the genetic diversity and population structure of eight cultured groups of *M. reevesii*, revealing critical insights for germplasm conservation and management. The resequencing data demonstrated high quality and alignment efficiency, enabling robust SNP-based analyses. Notably, the MM and WW populations exhibited the highest genetic diversity (PIC = 0.149 and 0.144; *Ho* = 0.299 and 0.287, respectively), likely due to effective management practices and heterozygous SNP enrichment. In contrast, the HZ and CH populations showed reduced diversity (lowest PIC and *Ho*) and elevated inbreeding coefficients (*F_HOM_* = 0.043 and 0.041), signalling risks of inbreeding depression from prolonged closed breeding. Despite overall low genetic differentiation (*F_st_* < 0.05), the MM and PX populations displayed distinct genetic clustering in PCA and phylogenetic analyses, suggesting unique ancestral origins or isolation-driven divergence. These populations may harbour adaptive alleles warranting prioritised conservation. To mitigate genetic erosion, targeted strategies are recommended: enhancing gene flow for inbred groups (e.g., HZ, CH) while preserving the genetic distinctiveness of MM and PX through controlled breeding. Future studies should integrate wild populations, employ long-read sequencing to resolve structural variants, and explore adaptive evolution mechanisms under environmental pressures. This work underscores the urgency of balancing aquaculture productivity with genetic diversity preservation to ensure the sustainability of *M. reevesii* germplasm resources.

## Figures and Tables

**Table 1:** Quality comparison of resequencing data of eight cultured groups of *M. reevesii*.

Group	Average Sequencing Number	Average Coverage (%)	Average Sequencing Depth (X)	Average Genome Mapping Rate (%)	GC Content (%)	Q30 (%)
JS	224,348,094	92.51	12.83	99.87	44.25	92.16
WW	233,554,144	92.53	13.36	99.87	44.30	93.28
ZQ	226,259,694	92.66	12.95	99.83	44.11	91.21
CH	211,544,440	92.30	11.83	98.54	44.08	92.20
PX	234,143,127	92.46	13.37	99.83	44.35	92.10
YY	218,711,083	92.40	12.51	99.83	44.07	92.46
MM	219,780,573	92.35	12.43	99.75	44.06	90.86
HZ	212,729,708	92.57	12.17	99.87	44.10	91.27

**Table 2:** Statistical analysis of SNP information for eight cultured groups of *M. reevesii*.

Group	ALL SNP	*Ho* (Homozygous)	*He* (Heterozygous)
JS	14,480,096	4,634,133 (32.36%)	9,845,963 (67.64%)
WW	14,473,593	4,593,932 (31.91%)	9,879,661 (68.09%)
ZQ	14,114,857	4,705,758 (33.35%)	9,409,099 (66.65%)
CH	15,505,440	4,705,754 (32.14%)	10,799,687 (67.86%)
PX	15,451,204	4,790,993 (31.45%)	10,660,212 (68.55%)
YY	14,364,542	4,712,537 (32.91%)	9,652,005 (67.09%)
MM	18,170,044	4,854,866 (26.94%)	13,315,177 (73.06%)
HZ	14,021,985	4,725,572 (33.73%)	9,296,413 (66.27%)

Legend: *Ho* = number of homozygous SNPs (percentage of total SNPs), *He* = number of heterozygous SNPs (percentage of total SNPs). Percentages were calculated as (*Ho* or *He* count/ALL SNP count) × 100.

**Table 3:** Genetic diversity of eight populations of *M. reevesii*.

Group	Nucleotide Diversity (π)	Inbreeding Coefficient (*F_HOM_*)	Polymorphism Information Content (PIC)	Observed Heterozygosity (*Ho*)
JS	0.0000373078	0.01050 ± 0.10593	0.14014 ± 0.01500	0.27958 ± 0.02993
WW	0.000037696	−0.01454 ± 0.11163	0.14379 ± 0.01581	0.28666 ± 0.03153
ZQ	0.000037056	0.03127 ± 0.02436	0.13721 ± 0.00353	0.27370 ± 0.00691
CH	0.0000369813	0.04064 ± 0.05698	0.13583 ± 0.00821	0.27099 ± 0.01627
PX	0.0000379257	0.00114 ± 0.06090	0.14147 ± 0.00849	0.28224 ± 0.01724
YY	0.0000372213	0.03081 ± 0.03405	0.13725 ± 0.00491	0.27382 ± 0.00966
MM	0.0000358555	−0.05729 ± 0.08055	0.14942 ± 0.01142	0.29875 ± 0.02280
HZ	0.0000369261	0.04325 ± 0.03725	0.13546 ± 0.00539	0.27028 ± 0.01060

Legend: Values for the inbreeding coefficient (*F_HOM_*), polymorphism information content (PIC), and observed heterozygosity (*Ho*) are presented as mean ± standard deviation.

**Table 4:** Pairwise population *F_st_* values among the eight populations of *M. reevesii*.

	WW	PX	ZQ	JS	CH	HZ	YY	MM
**WW**	—							
**PX**	0.00692	—						
**ZQ**	0.00104	0.00726	—					
**JS**	0.00119	0.00807	0.00205	—				
**CH**	0.00191	0.00608	0.00242	0.00193	—			
**HZ**	0.00093	0.00684	0.00043	0.00242	0.00266	—		
**YY**	0.00063	0.00545	0.00103	0.00181	0.00139	0.00081	—	
**MM**	0.04681	0.02500	0.04684	0.04758	0.04329	0.04608	0.04405	—

**Table 5:** The sample information of eight populations of *M. reevesii* used in this study.

Sampling Sites (Abbreviation Used)	Day/Month/Year	Sample Locality	GPS Coordinates	*N*
Wuwei (WW)	2 July 2021	Wuhu city, Anhui province	31°35′ N, 118°06′ E	30
Jingshan (JS)	5 November 2021	Jingshan City, Hubei Province	30°88′ N, 113°11′ E	30
Yiyang (YY)	1 November 2021	Yiyang County, Jiangxi Province	28°35′ N, 117°41′ E	28
Zhaoqing (ZQ)	29 October 2021	Zhaoqing City, Guangdong Province	22°97′ N, 112°63′ E	30
Huizhou (HZ)	28 October 2021	Huizhou City, Guangdong Province	22°86′ N, 113°68′ E	30
Maoming (MM)	25 October 2021	Maoming City, Guangdong Province	21°67′ N, 111°09′ E	30
Changhan (CH)	2 November 2021	Changde City, Hunan Province	28°84′ N, 111°82′ E	30
Pingxiang (PX)	9 November 2021	Pingxiang City, Guangxi Zhuang Autonomous region	22°16′ N, 106°86′ E	30

## Data Availability

The data presented in this study are available on request from the corresponding author. The data are not publicly available due to privacy or ethical restrictions.
